# Descriptive analysis of patients admitted to a new adolescent inpatient unit in Madrid

**DOI:** 10.1192/j.eurpsy.2022.1125

**Published:** 2022-09-01

**Authors:** M. Taracena Cuerda, M. Esperesate Pajares, M. Feito Garcia, C. Arranz Martin, E. Sánchez Sampedro, A.M. Jiménez Bidón, R. Puente García, C. Pastor Jordá

**Affiliations:** Hospital 12 de Octubre, Psychiatry, Madrid, Spain

**Keywords:** Adolescents, inpatient unit, suicidal

## Abstract

**Introduction:**

Adolescent mental health problems may have increased after COVID-19 worldwide pandemic. Therefore it seems necessary to study the state of mental health inpatient adolescent units.

**Objectives:**

Adolescent mental health problems may have increased after COVID-19 worldwide pandemic. Therefore it seems necessary to study the state of mental health inpatient adolescent units.

**Methods:**

An observational and descriptive analysis of the sample of patients between 12 and 17 years-old, that were admitted to the inpatient mental health unit since its opening on April 2021.

**Results:**

A total of 205 patients were admitted from April 2021 until October 2021. We have observed sex diferences within patients admitted, as the 82.9% of them were female. The mean age was 14.7, being 14.6 for girls and 15.3 for boys. The most common reason for admission (RFA) were suicidal ideation/attempt, eating disorders, affective disorders, conduct disorders/challenging behaviors and psychosis. Suicidal ideation/attempt was the most common RFA (57.07%) in both sexes, being higher among females (60.3%) than males (42.9%). Eating disorders were the second most common RFA in girls (17.7%) while psychosis (17.1%) and mood disorders (17.1%) were the second most common RFA within boys.

**Conclusions:**

Findings on how COVID-19 affected adolescents mental health are controversial in the literature, our data suggest that there is a need of developing quality studies that analyse how the pandemic might be influencing adolescents suicidal ideation/attempt and its protective and risk factors.

**Disclosure:**

No significant relationships.

